# Preoperative Risk Classification for Early Recurrence After Curative-Intent Resection of Perihilar Cholangiocarcinoma: A Single-Center Retrospective Study

**DOI:** 10.3390/cancers18213568

**Published:** 2026-11-06

**Authors:** Yu Fujimura, Naohisa Kuriyama, Yuki Segi, Haruna Komatsubara, Kazuyuki Gyoten, Takahiro Ito, Aoi Hayasaki, Takehiro Fujii, Yusuke Iizawa, Yasuhiro Murata, Akihiro Tanemura, Masashi Kishiwada, Shugo Mizuno

**Affiliations:** Department of Hepatobiliary Pancreatic and Transplant Surgery, Mie University Graduate School of Medicine, Tsu 514-8507, Japan; yu-fuji@med.mie-u.ac.jp (Y.F.); y-segi@med.mie-u.ac.jp (Y.S.); h-komatsu@med.mie-u.ac.jp (H.K.); k-gyoten@med.mie-u.ac.jp (K.G.); itotakahiro@med.mie-u.ac.jp (T.I.); a-hayasaki@med.mie-u.ac.jp (A.H.); t-fujii@med.mie-u.ac.jp (T.F.); uskm007@med.mie-u.ac.jp (Y.I.); yasumura@med.mie-u.ac.jp (Y.M.); iorichan@med.mie-u.ac.jp (A.T.); kishiwad@med.mie-u.ac.jp (M.K.); mizunos@med.mie-u.ac.jp (S.M.)

**Keywords:** perihilar cholangiocarcinoma, early recurrence, curative-intent resection, risk stratification, neutrophil-to-lymphocyte ratio, tumor markers

## Abstract

**Background/Objectives:** Early recurrence (ER) after curative-intent resection of perihilar cholangiocarcinoma (PHC) is associated with poor prognosis. However, simple preoperative predictors remain insufficiently defined. This study aimed to identify preoperative factors associated with ER and explore a risk classification. **Methods:** We retrospectively analyzed 108 patients who underwent curative-intent resection for PHC at a single institution between 2007 and 2023. ER was defined as recurrence within 1 year after surgery. Cox proportional hazards regression was used to identify associated factors. **Results:** ER occurred in 24 patients (22.2%). Multivariable analysis identified younger age (hazard ratio [HR] = 0.942), higher carcinoembryonic antigen (CEA; HR = 1.115), higher carbohydrate antigen 19-9 (CA19-9; HR = 1.001), and higher neutrophil-to-lymphocyte ratio (NLR; HR = 1.404) as factors associated with ER. In an exploratory classification, one point was assigned for age < 61 years, CEA ≥ 5.4 ng/mL, CA19-9 ≥ 89.4 U/mL, and NLR ≥ 2.037. Among 105 patients with complete data, the estimated 1-year recurrence rate was 70.0% in patients with 3–4 points (n = 10), versus 17.4% in those with 0–2 points (n = 95; *p* < 0.001). Bootstrap analysis suggested optimism in the score’s apparent discrimination when cutoff optimization was considered (optimism-corrected C-index, 0.668). **Conclusions:** Age, CEA, CA19-9, and NLR were associated with ER after PHC resection. The exploratory classification requires external validation before clinical use.

## Simple Summary

Perihilar cholangiocarcinoma is a bile duct cancer for which curative operation offers the main chance of long-term survival, but recurrence soon after surgery remains a major concern. This study aimed to identify factors available before surgery that could help predict early recurrence. We found that younger age, higher carcinoembryonic antigen and carbohydrate antigen 19-9 levels, and a higher neutrophil-to-lymphocyte ratio were associated with recurrence within one year after surgery. In an exploratory analysis, patients with three or more of these factors had an estimated 70% recurrence rate within one year, substantially higher than that of patients with two or fewer factors. Although these findings require validation in larger studies, this simple risk classification should be interpreted cautiously because the high-risk group included only 10 patients and should not guide clinical decisions without further validation. These findings may provide a basis for future studies investigating strategies to reduce recurrence and improve long-term outcomes.

## 1. Introduction

Perihilar cholangiocarcinoma (PHC) is a malignant tumor arising from the extrahepatic bile ducts near the hepatic hilum. Despite curative-intent resection, recurrence is frequent, and long-term outcomes remain unsatisfactory [1,2]. Early recurrence after curative-intent resection is associated with poor prognosis, and several studies have investigated its prognostic factors in PHC, including preoperatively assessable clinicopathological features [3,4,5].

More recently, prognostic models for recurrence after resection of PHC have been developed using multicenter datasets [6,7]. In addition, prediction of early recurrence has been investigated using large multi-institutional datasets and machine learning approaches. Specifically, Ratti et al. developed and validated a preoperative model to predict futile outcomes after upfront surgery, incorporating the risk of early recurrence among other adverse outcomes [8]. Wang et al. developed a machine learning-based model using multicenter data to predict early recurrence in patients with PHC planned for curative resection [9]. Kawashima et al. subsequently developed a machine learning-based model to predict very early recurrence within 6 months after curative-intent resection of PHC [10]. Although the definition of early recurrence varies among studies, recurrence within 1 year after surgery has been used to define early recurrence in PHC [3,4,5]. Therefore, we adopted a 1-year cutoff in the present study to maintain consistency with previous studies in PHC. However, these predictive models may require multiple variables and relatively complex calculations. Therefore, complementary approaches based on a limited number of readily available preoperative parameters may also be clinically useful.

In the present study, we defined early recurrence as recurrence within 1 year after curative-intent resection and sought to identify readily assessable preoperative factors associated with early recurrence, with a particular focus on tumor markers and immune–nutritional and inflammatory markers, and to explore a simple approach for identifying patients at particularly high risk of early recurrence. We identified four preoperatively assessable predictors of early recurrence and developed a simple risk classification that identified patients at high risk of recurrence within 1 year after surgery.

## 2. Materials and Methods

### 2.1. Patient Selection and Ethical Approval

**Figure 1:**
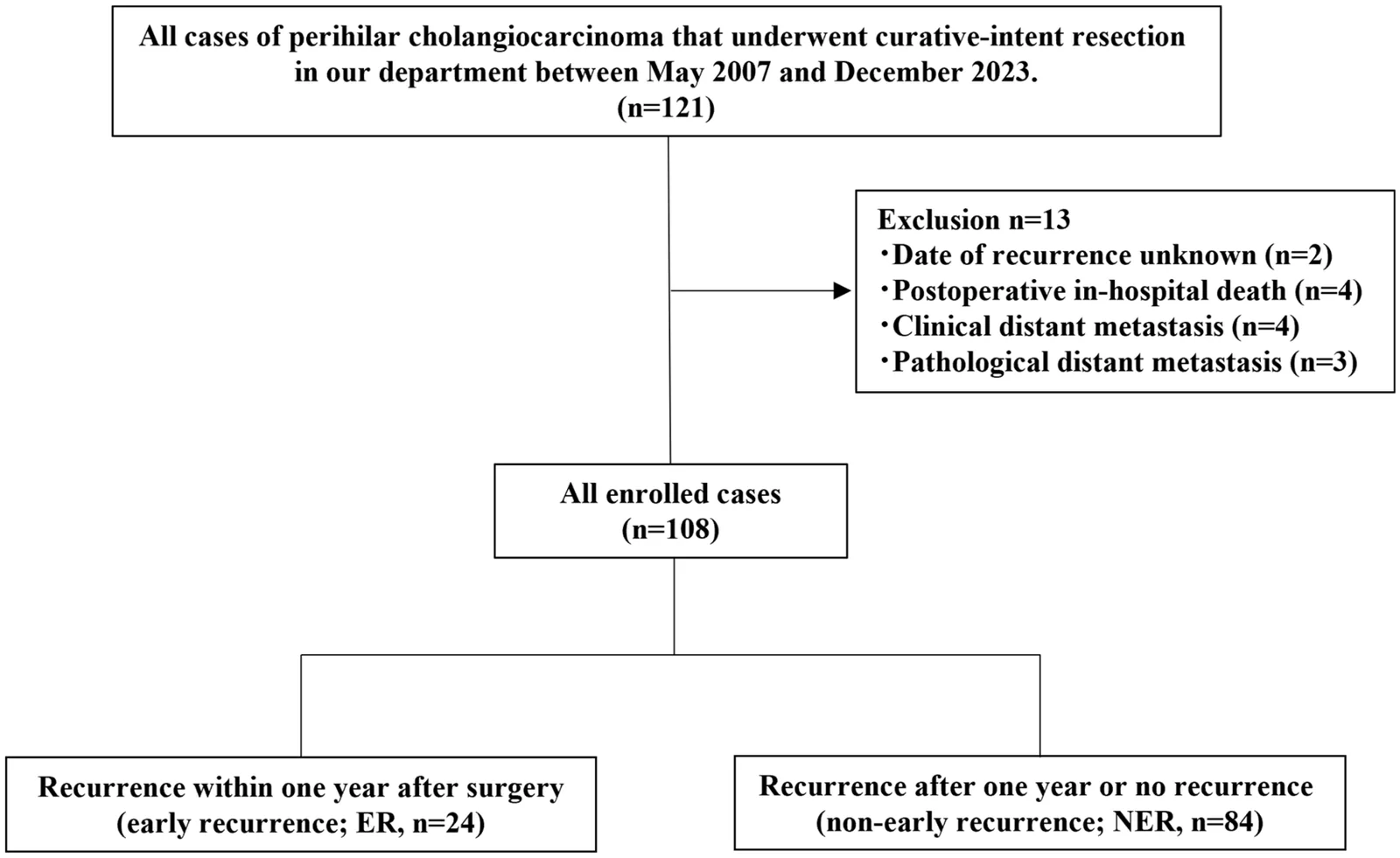
The study flow diagram.

Among 169 patients with PHC treated at the Department of Hepatobiliary Pancreatic and Transplant Surgery of Mie University Hospital between May 2007 and December 2023, 121 underwent curative-intent resection. As shown in Figure 1, 108 patients were included in the present study after excluding 13 patients from the resected cohort. Of the 13 excluded patients, four died during the postoperative hospital stay before recurrence could be assessed and were therefore excluded from the analysis. The 108 included patients were classified into the early recurrence (ER) group (n = 24), defined as recurrence within 1 year after surgery, and the non-early recurrence (NER) group (n = 84), comprising patients with recurrence more than 1 year after surgery or no recurrence.

This study was conducted in accordance with the ethical standards of the Declaration of Helsinki and its later amendments and was approved by the Institutional Review Board of Mie University, Japan (approval number H2018-64; approval date: 29 October 2018). Informed consent was obtained through an opt-out procedure.

### 2.2. Preoperative Management

Preoperative diagnosis and staging were performed using multidetector-row computed tomography (MDCT), magnetic resonance imaging/magnetic resonance cholangiopancreatography (MRI/MRCP), endoscopic retrograde cholangiography (ERC), and intraductal ultrasonography (IDUS). The local extent of tumor spread was further assessed using IDUS and cholangioscopy. In patients with obstructive jaundice, biliary drainage was performed using endoscopic retrograde biliary drainage (ERBD) or endoscopic nasobiliary drainage (ENBD), with percutaneous transhepatic biliary drainage (PTBD) used in selected cases. Preoperative laboratory parameters, including tumor markers and inflammatory indices, were assessed using blood samples obtained immediately before surgery. In patients who received neoadjuvant therapy, laboratory values obtained after completion of neoadjuvant therapy and before surgery were used for analysis. In patients who underwent biliary drainage, laboratory values obtained after drainage were used. Surgery was generally performed after clinical resolution of cholangitis, although inflammatory markers were not necessarily normalized in all patients.

### 2.3. Neoadjuvant Therapy

Until 2010, the indication for preoperative treatment was determined on a case-by-case basis, considering the extent of local progression and lymph node enlargement. Chemoradiotherapy (CRT) was primarily selected, combining gemcitabine with 35 Gy or 45 Gy of local radiation.

Since 2011, neoadjuvant chemotherapy (NAC) has been administered in cases diagnosed with preoperative lymph node metastasis, cases requiring combined resection and reconstruction of the portal vein (PV) and/or hepatic artery (HA), cases in which PV and HA resection or reconstruction were not feasible, and cases where resection was deemed impossible even with left or right trisectionectomy. The NAC regimen consisted of two cycles of gemcitabine (600 mg/m^2^ on days 7 and 21) in combination with S-1 (60 mg/m^2^ daily on days 1–21 in a 4-week cycle) [11].

### 2.4. Surgical Procedure

Right hepatectomy with caudate lobectomy was mainly indicated for Bismuth types I, II, and IIIa, and left hepatectomy with caudate lobectomy for type IIIb. For type IV, trisectionectomy was performed when R0 resection was deemed feasible. Liver resection was omitted in type I cases curatively treated by pancreatoduodenectomy (PD) alone. The final choice of hepatectomy was guided by the possibility of R0 resection based on imaging, integrating the future liver remnant (FLR) ratio with indocyanine green plasma clearance to assess FLR function. The FLR ratio was calculated using MDCT and 99mTc-GSA scintigraphy (15 min uptake), analyzed with 3D software (Synapse Vincent, Fujifilm, Tokyo, Japan). Resections with a functional FLR value < 0.05 were avoided due to the high risk of liver failure. Preoperative portal vein embolization was performed when the predicted FLR volume was <40% or the functional value was <0.05.

### 2.5. Immune–Nutritional and Inflammatory Markers

The values were calculated from blood test results obtained immediately before surgery. The neutrophil-to-lymphocyte ratio (NLR), monocyte-to-lymphocyte ratio (MLR), and platelet-to-lymphocyte ratio (PLR) were defined as the respective absolute cell counts divided by the lymphocyte count. The C-reactive protein-to-albumin ratio (CAR) was expressed as the ratio of serum C-reactive protein (CRP; mg/dL) to albumin (g/dL). The prognostic nutritional index (PNI) was determined according to Onodera et al. using the equation 10 × serum albumin (g/dL) + 0.005 × total lymphocyte count (/μL) [12].

Nutritional status was also assessed using the Controlling Nutritional Status (CONUT) score, which combines three components: serum albumin, total lymphocyte count, and total cholesterol [13]. Albumin concentrations of ≥3.50, 3.00–3.49, 2.50–2.99, and <2.50 g/dL were assigned 0, 2, 4, and 6 points, respectively. Lymphocyte counts of ≥1600, 1200–1599, 800–1199, and <800/μL were assigned 0, 1, 2, and 3 points, respectively, whereas cholesterol concentrations of ≥180, 140–179, 100–139, and <100 mg/dL corresponded to 0, 1, 2, and 3 points, respectively. The three component scores were added to obtain the CONUT score.

The C-reactive protein–albumin–lymphocyte (CALLY) index was derived from serum albumin, lymphocyte count, and CRP according to the following equation: [albumin (g/dL) × lymphocyte count (/μL)]/[10,000 × CRP (mg/dL)] [14].

The systemic immune–inflammation index (SII) was derived from the neutrophil, platelet, and lymphocyte counts using the formula [neutrophil count (/μL) × platelet count (/μL)]/[lymphocyte count (/μL) × 1000] [15]. The systemic inflammation response index (SIRI) was obtained from the neutrophil, monocyte, and lymphocyte counts using the formula [neutrophil count (/μL) × monocyte count (/μL)]/[lymphocyte count (/μL) × 1000] [16].

### 2.6. Adjuvant Chemotherapy and Follow-Up

Postoperative adjuvant chemotherapy was generally initiated 4–6 weeks after surgery, with a planned treatment duration of at least 6 months. The institutional regimen was changed in June 2013. Before this change, gemcitabine was administered at 800 mg/m^2^ every 2 weeks. Thereafter, oral S-1 was used at 60 mg/m^2^/day for 28 consecutive days, followed by a 14-day drug-free interval in each 42-day cycle.

Postoperative surveillance consisted of tumor marker evaluation every 2 to 3 months and contrast-enhanced CT every 4 to 6 months. If tumor marker levels increased, contrast-enhanced CT was performed immediately. Postoperative recurrence was diagnosed based on imaging findings. The final follow-up date was 1 January 2025. The median follow-up duration was 64.4 months.

### 2.7. Statistical Analysis

Continuous data were summarized using the median and range, and between-group differences were evaluated with the Mann–Whitney U test. For categorical data, the number and percentage of patients in each category were reported, with comparisons performed using either the chi-square test or Fisher’s exact test, depending on the data. Associations between candidate variables and early recurrence were examined through Cox proportional hazards regression analysis. For the Cox analyses, the event of interest was the first postoperative recurrence occurring within 12 months after curative-intent surgery. Time was measured in months from the date of surgery, and follow-up was administratively censored at 12 months. Recurrences occurring after 12 months were not counted as events. Patients who died without recurrence were censored at the date of death, whereas those lost to follow-up were censored at the date of their last confirmed recurrence-free assessment. Following univariable screening, variables showing a significant association with early recurrence (age, CEA, CA19-9, NLR, PLR, SII, SIRI, and clinical T classification) were entered as candidate covariates into a multivariable Cox proportional hazards model using a backward stepwise selection procedure based on Wald test *p* values. Multicollinearity was assessed using variance inflation factors (VIFs) for the eight candidate variables and the four variables retained in the final model. Cutoff values for age, CEA, CA19-9, and NLR were determined using receiver operating characteristic (ROC) curve analysis based on the Youden index. To assess the impact of missing NLR values, a complete-case sensitivity analysis was performed after excluding three patients with missing NLR data. The four-factor risk classification was reassessed in patients with complete data for age, CEA, CA19-9, and NLR. In addition, a multivariable Cox proportional hazards model including these four predictors was fitted without stepwise variable selection.

**Figure 2:**
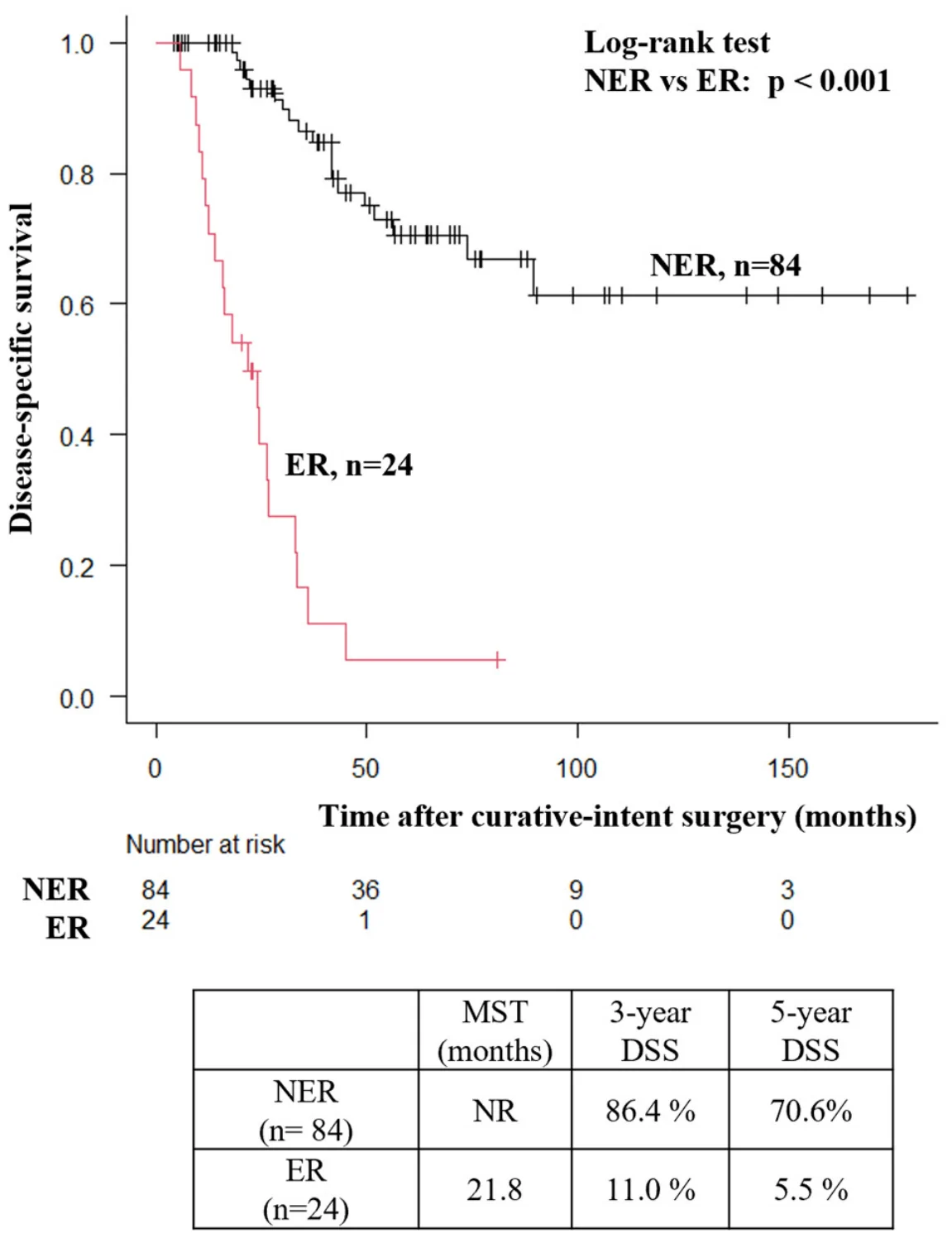
Kaplan–Meier analysis of disease-specific survival (DSS) according to early recurrence status after curative-intent surgery. Patients were classified into the early recurrence (ER) and non-early recurrence (NER) groups based on recurrence within 12 months after surgery. DSS was calculated from the date of surgery. The comparison between the ER and NER groups was descriptive because group classification was based on postoperative recurrence status. MST, median survival time; NR, not reached.

**Figure 3:**
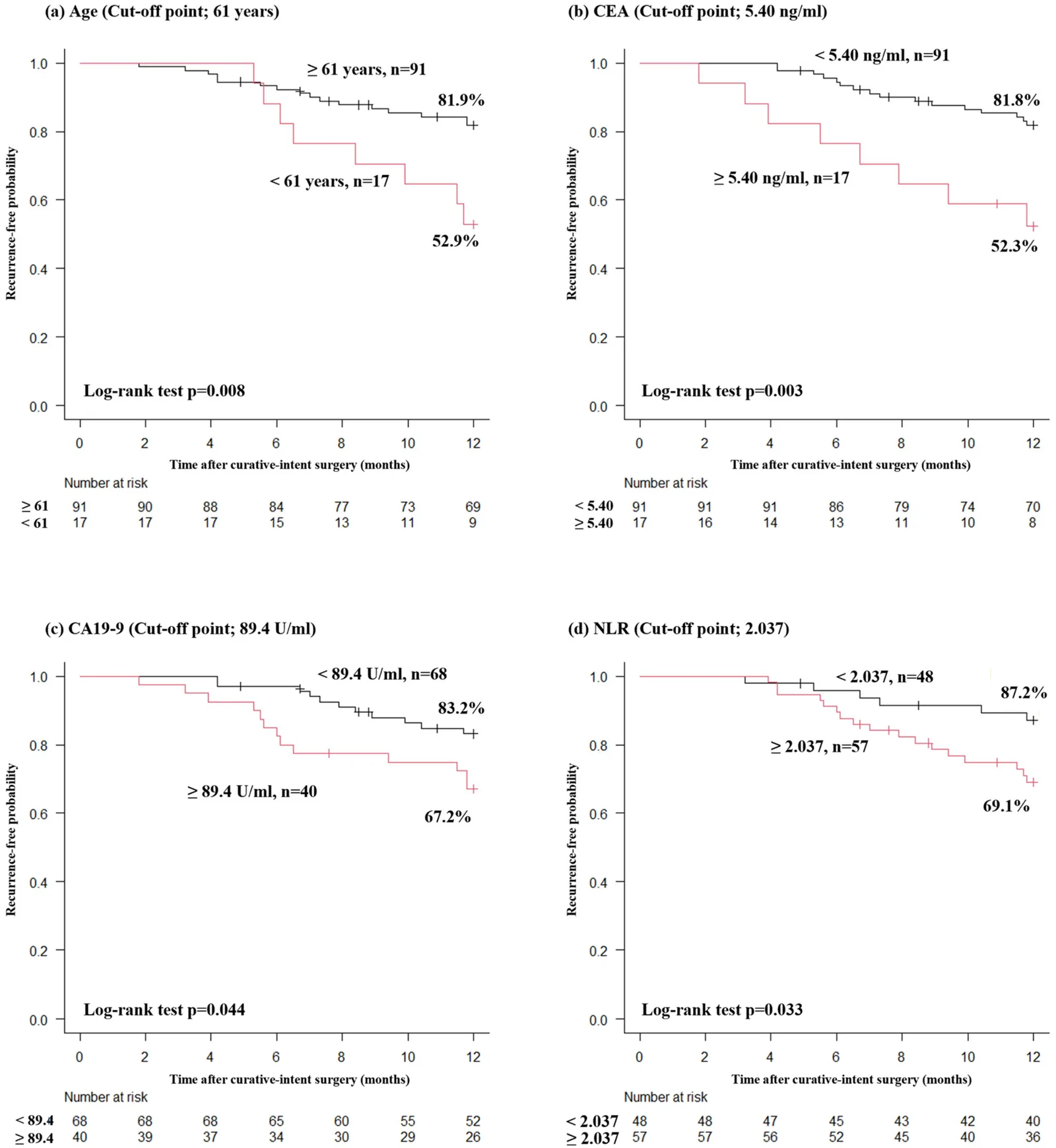
Kaplan–Meier analysis of recurrence-free probability during the first 12 months after surgery according to cutoff values of preoperative risk factors. (**a**) Age, <61 vs. ≥61 years; (**b**) CEA, <5.4 vs. ≥5.4 ng/mL; (**c**) CA19-9, <89.4 vs. ≥89.4 U/mL; and (**d**) NLR, <2.037 vs. ≥2.037. CEA, carcinoembryonic antigen; CA19-9, carbohydrate antigen 19-9; NLR, neutrophil-to-lymphocyte ratio. NLR data were available for 105 patients.

**Figure 4:**
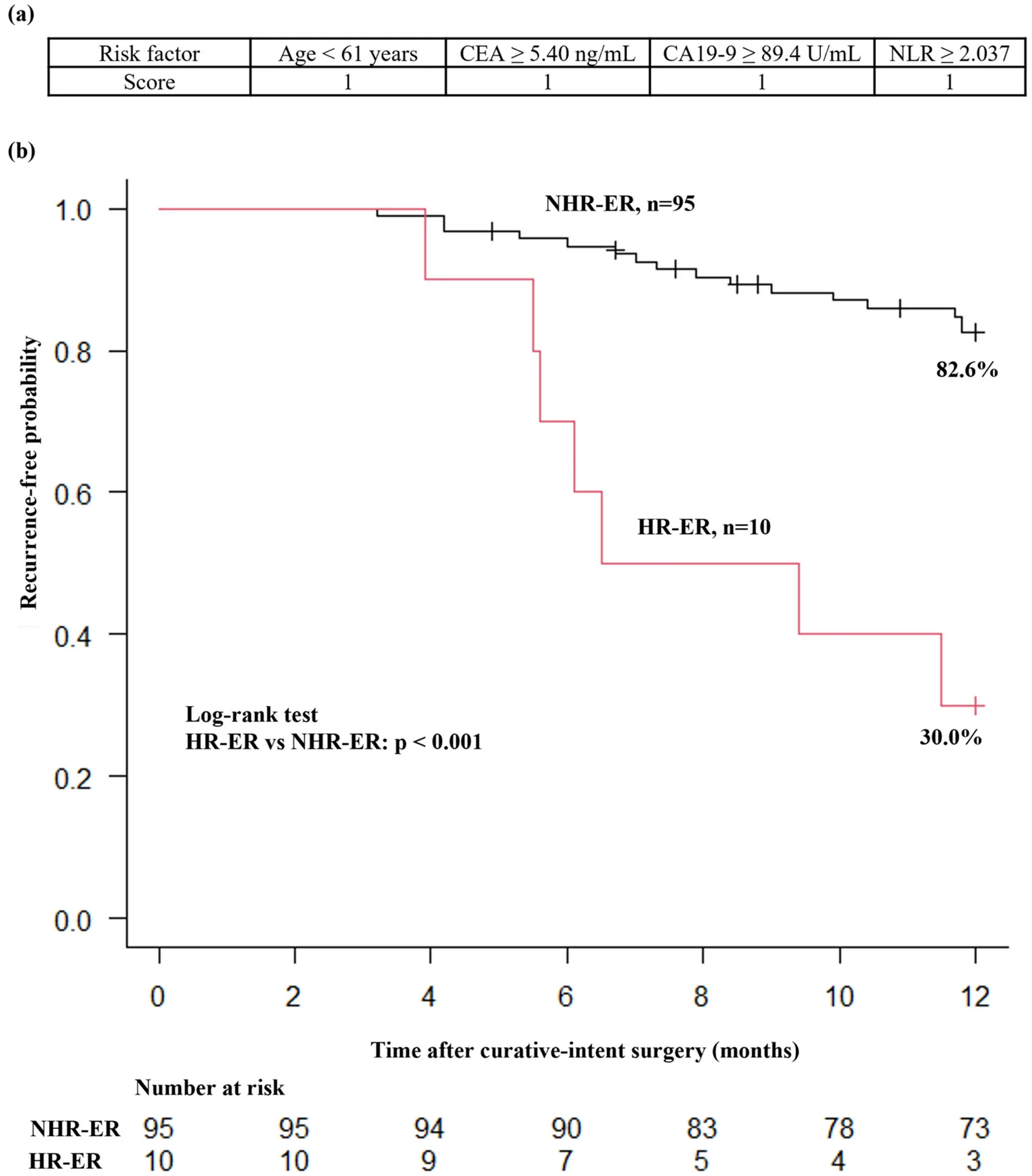
Risk classification for early recurrence based on preoperative risk factors. (**a**) A scoring system was developed by assigning 1 point for each of the following risk factors: age < 61 years, CEA ≥ 5.4 ng/mL, CA19-9 ≥ 89.4 U/mL, and NLR ≥ 2.037. Patients with 3–4 points were classified as the HR-ER group, whereas those with 0–2 points were classified as the NHR-ER group. (**b**) Kaplan–Meier analysis of recurrence-free probability during the first 12 months after surgery in the HR-ER and NHR-ER groups. Three patients with missing NLR data were excluded from the analysis (n = 105). CEA, carcinoembryonic antigen; CA19-9, carbohydrate antigen 19-9; NLR, neutrophil-to-lymphocyte ratio; HR-ER, high-risk early recurrence; NHR-ER, non-high-risk early recurrence.

**Figure 5:**
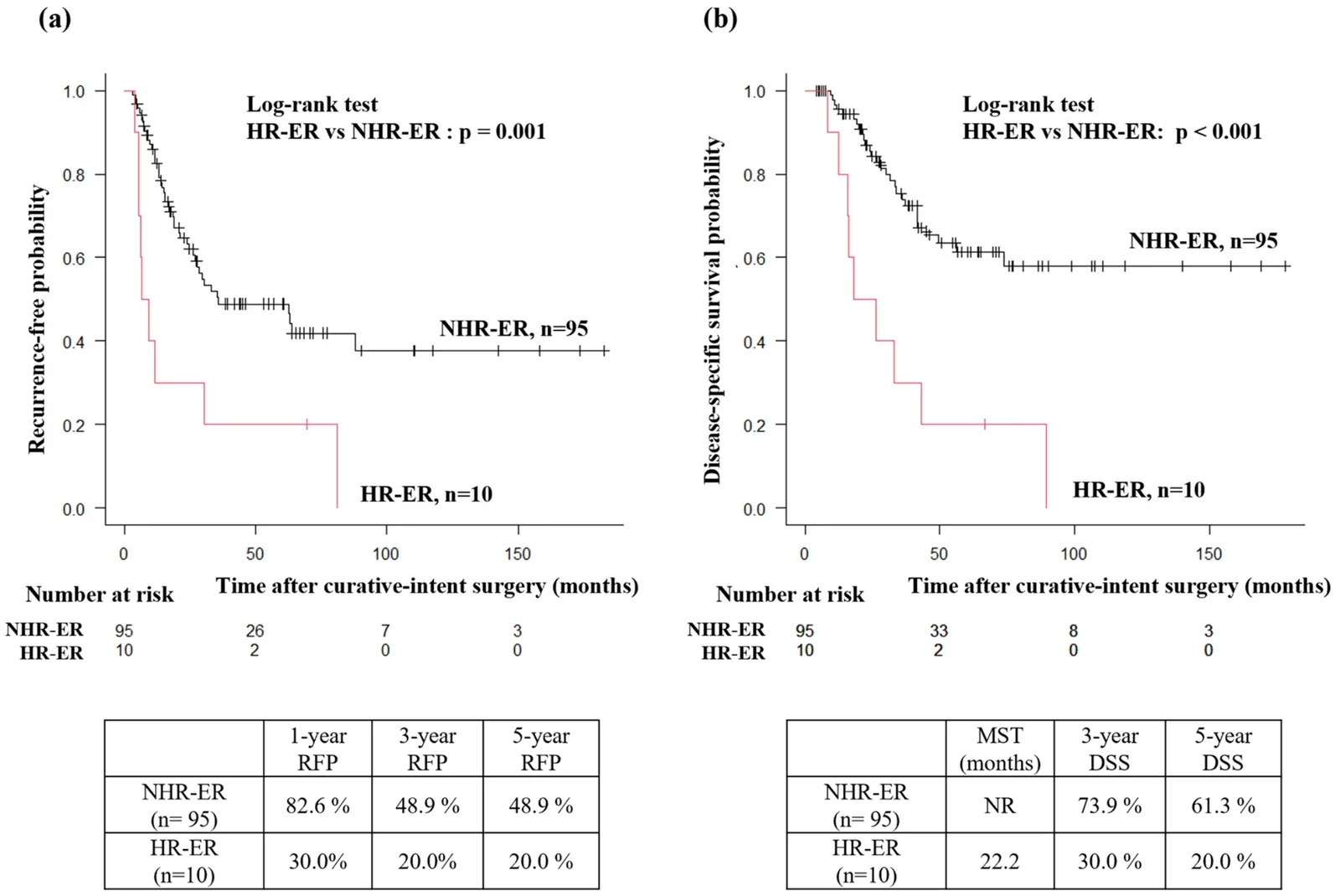
Kaplan–Meier analysis of RFP and DSS according to early recurrence risk groups. (**a**) RFP in the HR-ER and NHR-ER groups. (**b**) DSS after curative-intent surgery in the HR-ER and NHR-ER groups. The analyses included 105 patients with complete data for the four preoperative risk factors. Numbers at risk are shown below the Kaplan–Meier curves. RFP, recurrence-free probability; DSS, disease-specific survival; HR-ER, high-risk early recurrence; NHR-ER, non-high-risk early recurrence; MST, median survival time; NR, not reached.

The Kaplan–Meier method was applied to estimate the 1-year recurrence rate and recurrence-free probability (RFP) over the entire follow-up period, and disease-specific survival (DSS) was also estimated using the Kaplan–Meier method. For recurrence-related Kaplan–Meier analyses, postoperative recurrence alone was defined as the event, and recurrence-free probability represented the Kaplan–Meier estimate of remaining free from recurrence, rather than a composite endpoint of recurrence or death. Figure 2 evaluated DSS, Figure 3 and Figure 4 evaluated early recurrence within the first 12 postoperative months, and Figure 5 evaluated recurrence over the entire follow-up period. Between-group comparisons of survival curves were performed with the log-rank test. Median follow-up time was determined by applying the reverse Kaplan–Meier approach. For the analysis presented in Figure 4, patients were divided into two categories based on their total number of risk factors: three or four versus zero, one, or two.

The stability of variable selection was further explored using 1000 bootstrap resamples, in which backward stepwise selection based on Wald test *p* values was repeated among the eight candidate variables. The frequency with which each variable was retained was calculated across the bootstrap resamples.

To assess potential optimism in the performance of the exploratory four-factor risk classification, bootstrap analyses with 1000 resamples were performed. In the first analysis, the original cutoff values were held fixed, and optimism in the C-index was estimated by comparing performance in the bootstrap samples with that in the original dataset. In a separate analysis, cutoff values for the four prespecified predictors were re-estimated within each bootstrap sample using ROC curve analysis based on the Youden index. Optimism-corrected discrimination was assessed using the C-index, and calibration was evaluated using the calibration slope. These analyses did not repeat predictor selection and optimization of the score-grouping threshold within a single bootstrap procedure.

Statistical significance was defined as a two-sided *p* value below 0.05. The analyses were conducted with EZR software (version 1.68; Saitama Medical Center, Jichi Medical University, Saitama, Japan) [17]. R scripts for selected statistical analyses, including bootstrap internal validation, were developed with the assistance of generative artificial intelligence (AI).

## 3. Results

### 3.1. Disease-Specific Survival According to Early Recurrence Status

As shown in Figure 2, DSS at 3 and 5 years in the NER group was estimated at 86.4% (95% CI, 75.5–92.7%) and 70.6% (95% CI, 56.6–80.8%), respectively. The corresponding estimates in the ER group were 11.0% (95% CI, 1.9–29.3%) and 5.5% (95% CI, 0.4–22.1%). Although the median DSS had not been reached in the NER group, it was 21.8 months in the ER group. The difference in DSS between the two groups was statistically significant, with poorer survival observed in the ER group (*p* < 0.001). Because the ER and NER groups were defined according to postoperative recurrence status, this comparison was considered descriptive.

### 3.2. Preoperative Characteristics of the ER and NER Groups

Patient characteristics are shown in Table 1. The median age was 66 years (range, 39–83 years) in the ER group and 71 years (range, 44–87 years) in the NER group, with patients in the NER group being significantly older (*p* = 0.015). The ER group comprised 15 male and 9 female patients, whereas the NER group comprised 59 male and 25 female patients. There was no significant difference in sex distribution between the two groups (*p* = 0.472).

There were no significant differences between the two groups in preoperative management, major laboratory parameters, or immune–nutritional and inflammatory markers. Clinical T classification was significantly more advanced in the ER group than in the NER group (*p* = 0.002), whereas no significant difference was observed in clinical N classification (*p* = 0.348).

Surgical characteristics, resection margin status, postoperative complications, adjuvant chemotherapy, and recurrence patterns in the ER and NER groups are summarized in Table S1.

### 3.3. Univariable and Multivariable Analyses of Early Recurrence

Table 2 summarizes the results of the univariable and multivariable Cox proportional hazards models used to examine predictors of early recurrence following curative-intent resection for PHC.

In the univariable analysis, age was significantly associated with early recurrence (hazard ratio [HR] = 0.951, 95% confidence interval [CI]: 0.916–0.989, *p* = 0.011), whereas sex, with male sex as the reference, was not significantly associated with early recurrence (HR = 1.438, 95% CI: 0.629–3.287, *p* = 0.389). No variables related to preoperative management or routine laboratory parameters were significantly associated with early recurrence. In contrast, the tumor markers CEA (HR = 1.104, 95% CI: 1.057–1.153, *p* < 0.001) and CA19-9 (HR = 1.001, 95% CI: 1.000–1.001, *p* < 0.001) were significantly associated with early recurrence. Among the immune–nutritional and inflammatory markers, NLR, PLR, SII, and SIRI were significantly associated with early recurrence (NLR: HR = 1.406, 95% CI: 1.068–1.850, *p* = 0.015; PLR: HR = 1.006, 95% CI: 1.002–1.009, *p* = 0.003; SII: HR = 1.001, 95% CI: 1.000–1.002, *p* = 0.003; SIRI: HR = 1.447, 95% CI: 1.008–2.076, *p* = 0.045). Regarding clinical tumor classification, advanced clinical T classification (cT3 or higher) was significantly associated with early recurrence (HR = 5.791, 95% CI: 1.361–24.628, *p* = 0.017), whereas clinical N classification was not (HR = 1.544, 95% CI: 0.661–3.608, *p* = 0.316).

In the multivariable analysis, all variables significantly associated with early recurrence in the univariable analysis (age, CEA, CA19-9, NLR, PLR, SII, SIRI, and clinical T classification) were entered as candidate covariates into a Cox proportional hazards model using a backward stepwise selection procedure based on Wald test *p* values. Age, CEA, CA19-9, and NLR were retained in the final model, whereas PLR, SII, SIRI, and clinical T classification were removed during the stepwise selection process. Younger age, higher CEA, higher CA19-9, and higher NLR were identified as independent predictors of early recurrence following curative-intent resection (age: HR = 0.942, 95% CI: 0.901–0.985, *p* = 0.008; CEA: HR = 1.115, 95% CI: 1.056–1.177, *p* < 0.001; CA19-9: HR = 1.001, 95% CI: 1.000–1.001, *p* = 0.024; NLR: HR = 1.404, 95% CI: 1.035–1.905, *p* = 0.029).

Multicollinearity assessment showed elevated variance inflation factors (VIFs) for SII (8.798) and PLR (4.986), whereas the VIFs for the four variables retained in the final model ranged from 1.019 to 1.134 (Table S2).

In an exploratory bootstrap analysis of variable selection using 1000 resamples, the selection frequencies were 56.3% for age, 79.1% for CEA, 53.5% for CA19-9, and 39.9% for NLR, suggesting considerable variability in predictor selection.

To assess the impact of missing NLR values, an additional multivariable Cox proportional hazards analysis was performed in 105 patients with complete data for age, CEA, CA19-9, and NLR, including 23 patients with early recurrence. All four predictors were included without stepwise variable selection. Age (HR = 0.936, 95% CI: 0.895–0.978, *p* = 0.003), CEA (HR = 1.121, 95% CI: 1.062–1.184, *p* < 0.001), CA19-9 (HR = 1.0005, 95% CI: 1.0001–1.0010, *p* = 0.022), and NLR (HR = 1.362, 95% CI: 1.003–1.851, *p* = 0.048) remained significantly associated with early recurrence. These findings were consistent with those of the original multivariable analysis (Table S3).

To further investigate the association between younger age and early recurrence, an exploratory comparison of clinicopathological characteristics was performed between patients aged < 61 years (n = 17) and those aged ≥ 61 years (n = 91). Younger patients had significantly higher rates of neoadjuvant therapy (76.5% vs. 45.1%, *p* = 0.032) and portal vein resection and reconstruction (64.7% vs. 34.1%, *p* = 0.028), as well as a significantly different distribution of pathological T classification (*p* = 0.004). The neutrophil-to-lymphocyte ratio was also significantly higher in younger patients (median, 2.66 vs. 2.02, *p* = 0.039). The detailed results are presented in Table S4.

### 3.4. Recurrence-Free Probability According to Risk Factors

The independent predictors of early recurrence after curative-intent resection identified by multivariable analysis were younger age, higher CEA, higher CA19-9, and higher NLR. Optimal cutoff values for these factors were determined by receiver operating characteristic (ROC) curve analysis using the Youden index: 61 years for age, 5.4 ng/mL for CEA, 89.4 U/mL for CA19-9, and 2.037 for NLR. As shown in Figure 3, Kaplan–Meier curves for recurrence-free probability during the first 12 months after surgery were compared according to these cutoff values. Patients aged < 61 years, with CEA ≥ 5.4 ng/mL, CA19-9 ≥ 89.4 U/mL, or NLR ≥ 2.037 had significantly lower recurrence-free probabilities. The corresponding 1-year recurrence rates were 47.1% versus 18.1% for age (*p* = 0.008), 47.7% versus 18.2% for CEA (*p* = 0.003), 32.8% versus 16.8% for CA19-9 (*p* = 0.044), and 30.9% versus 12.8% for NLR (*p* = 0.033). The corresponding 1-year recurrence-free probabilities (95% CIs) were 52.9% (27.6–73.0%) versus 81.9% (72.1–88.5%) for age, 52.3% (26.8–72.7%) versus 81.8% (72.1–88.5%) for CEA, 67.2% (50.2–79.4%) versus 83.2% (71.7–90.3%) for CA19-9, and 69.1% (55.1–79.6%) versus 87.2% (73.6–94.0%) for NLR.

### 3.5. High-Risk Group for Early Recurrence After Surgery

Each of the four independent predictors of early recurrence was assigned 1 point: age < 61 years, CEA ≥ 5.4 ng/mL, CA19-9 ≥ 89.4 U/mL, and NLR ≥ 2.037. Three patients with missing NLR data were excluded from the risk classification analysis, leaving 105 patients for evaluation. The numbers of patients with 4, 3, 2, 1, and 0 points were 1, 9, 28, 42, and 25, respectively.

Kaplan–Meier analysis of recurrence-free probability during the first 12 months after surgery showed similar patterns among patients with 0–2 points, whereas patients with 3 points showed a lower recurrence-free probability. Because only one patient had 4 points, the recurrence pattern of this subgroup could not be meaningfully evaluated separately. Based on these observed recurrence patterns, patients were subsequently classified into two groups according to the number of risk factors: those with 0–2 points were defined as the non-high-risk early recurrence (NHR-ER) group, whereas those with 3–4 points were defined as the high-risk early recurrence (HR-ER) group. The NHR-ER and HR-ER groups comprised 95 and 10 patients, respectively. As shown in Figure 4, the 1-year recurrence rate was 17.4% (95% CI: 11.1–26.9%) in the NHR-ER group and 70.0% (95% CI: 42.2–92.9%) in the HR-ER group (*p* < 0.001).

Exploratory sensitivity analyses were performed to assess the potential influence of neoadjuvant therapy (NAT), preoperative cholangitis, and surgical era. Among patients who did not receive NAT (n = 54), the estimated 1-year recurrence rates were 75.0% in the HR-ER group and 14.5% in the NHR-ER group (*p* < 0.001). Among patients without preoperative cholangitis (n = 53), the corresponding rates were 100% and 20.4%, respectively (*p* < 0.001). In an exploratory subgroup analysis stratified by surgical era, the 1-year recurrence rates in the HR-ER and NHR-ER groups were 50.0% and 21.8%, respectively, during 2007–2015 (*p* = 0.20), and 100% and 14.4%, respectively, during 2016–2023 (*p* < 0.001) (Table S5). These findings should be interpreted cautiously because of the very small number of HR-ER patients in each subgroup.

In an exploratory bootstrap analysis using 1000 resamples, the apparent C-index was 0.723 and the optimism-corrected C-index was 0.720 when the original cutoff values were held fixed. In a separate bootstrap analysis in which the cutoff values for the four prespecified predictors were re-estimated within each resample, the apparent C-index was 0.743, and the optimism-corrected C-index was 0.668. The optimism-corrected calibration slope in the latter analysis was 0.752.

### 3.6. Clinicopathological Features of the HR-ER and NHR-ER Groups

The clinicopathological characteristics of the HR-ER and NHR-ER groups are summarized in Table 3. Among the risk factors for early recurrence, age, CA19-9, and NLR differed significantly between the two groups, with the HR-ER group having a younger age (*p* = 0.007) and higher CA19-9 (*p* < 0.001) and NLR (*p* = 0.002) levels. In contrast, CEA levels did not differ significantly between the two groups (*p* = 0.095). Regarding other preoperative factors, the HR-ER group had significantly higher WBC counts (*p* = 0.038), neutrophil counts (*p* = 0.013), MLR (*p* = 0.037), PLR (*p* = 0.012), SII (*p* = 0.001), and SIRI (*p* = 0.005). Among intraoperative, postoperative, and pathological factors, portal vein resection and reconstruction was more frequently performed in the HR-ER group (*p* = 0.041), and the HR-ER group had significantly greater blood loss (*p* = 0.005) and a higher frequency of lymph node metastasis (*p* = 0.001). No significant difference in pathological T classification was observed between the two groups (*p* = 0.134).

### 3.7. RFP and DSS According to Early Recurrence Risk Groups

The RFP of the HR-ER and NHR-ER groups and the DSS of the same two groups are shown in Figure 5. Regarding RFP, the 1-, 3-, and 5-year RFP values were 30.0%, 20.0% (95% CI: 3.1–47.5%), and 20.0% (95% CI: 3.1–47.5%), respectively, in the HR-ER group, compared with 82.6%, 48.9% (95% CI: 37.3–59.4%), and 48.9% (95% CI: 37.3–59.4%), respectively, in the NHR-ER group. RFP was significantly lower in the HR-ER group than in the NHR-ER group (*p* = 0.001). Regarding DSS after curative-intent surgery, the 3- and 5-year DSS rates were 73.9% (95% CI: 62.3–82.4%) and 61.3% (95% CI: 48.3–72.0%), respectively, in the NHR-ER group, compared with 30.0% (95% CI: 7.1–57.8%) and 20.0% (95% CI: 3.1–47.5%), respectively, in the HR-ER group. The median DSS was not reached in the NHR-ER group and was 22.2 months in the HR-ER group. DSS was significantly lower in the HR-ER group than in the NHR-ER group (*p* < 0.001).

## 4. Discussion

In patients with PHC undergoing curative-intent resection, early postoperative recurrence was associated with unfavorable prognosis. Our analysis identified four preoperatively assessable factors independently associated with early recurrence: younger age, elevated CEA, elevated CA19-9, and elevated NLR. In an exploratory analysis, we further classified patients according to the number of these factors, using thresholds of age < 61 years, CEA ≥ 5.4 ng/mL, CA19-9 ≥ 89.4 U/mL, and NLR ≥ 2.037. Patients with three or more factors were assigned to the HR-ER group, while those with two or fewer constituted the NHR-ER group. The estimated 1-year recurrence-free probability was 30.0% in the HR-ER group, compared with 82.6% in the NHR-ER group (*p* < 0.001). The HR-ER group also showed a lower recurrence-free probability over the entire follow-up period (*p* = 0.001) and poorer DSS (*p* < 0.001).

NLR, calculated from neutrophil and lymphocyte counts obtained from routine blood tests, is a readily available systemic inflammatory marker that has been reported to be associated with prognosis in various malignancies [18,19]. Hu et al. reported that preoperative NLR was useful for predicting early postoperative recurrence in patients with perihilar cholangiocarcinoma [20]. More recently, Guo et al. also reported that preoperative NLR was independently associated with early recurrence after resection of hilar cholangiocarcinoma [21]. Consistent with this finding, NLR was identified as an independent predictor of early recurrence after curative-intent resection in the present study. Furthermore, patients with an NLR ≥ 2.037 had a significantly higher 1-year recurrence rate than those with an NLR < 2.037 (30.9% vs. 12.8%, *p* = 0.033), supporting the potential utility of preoperative NLR for identifying patients at increased risk of early recurrence. The difference in NLR cutoff values between studies may reflect differences in patient populations and cutoff determination methods, highlighting the need for external validation of the optimal cutoff for predicting early recurrence in PHC.

In the present study, both CEA and CA19-9 were identified as independent predictors of early recurrence after curative-intent resection. The prognostic significance of preoperative tumor markers, including CEA and CA19-9, in patients undergoing resection for PHC has been reported previously [22]. Kuriyama et al. reported that elevated preoperative CEA was associated with poorer DSS after curative-intent resection [23]. Regarding early recurrence, Yamamoto et al. and Hu et al. reported an association between elevated CA19-9 levels and early recurrence [4,20]. Our findings further suggest that preoperative CEA, in addition to CA19-9, may be useful for identifying patients at increased risk of early recurrence after resection of PHC.

Interestingly, younger age was independently associated with early recurrence in the present study, although advanced age is generally associated with poorer outcomes in many malignancies. One possible clinical explanation is that younger patients may have been more likely to receive intensive multidisciplinary treatment and undergo aggressive surgical resection for locally advanced disease. In an exploratory age-stratified analysis, patients younger than 61 years had significantly higher rates of neoadjuvant therapy (76.5% vs. 45.1%, *p* = 0.032) and portal vein resection (64.7% vs. 34.1%, *p* = 0.028) than older patients, as well as a significantly different distribution of pathological T classification (*p* = 0.004; Table S4). These findings suggest that differences in disease characteristics and treatment selection may have contributed to the observed association between younger age and early recurrence. Differences in tumor biology may also play a role, although this possibility could not be evaluated in the present study. Given the small number of younger patients and the retrospective design, these explanations remain speculative and warrant further investigation.

In the present study, age, CEA, CA19-9, and NLR were identified as independent predictors of early recurrence, and each factor was assigned 1 point for risk stratification. CEA and CA19-9 levels may be elevated in the presence of biliary obstruction or cholangitis [24,25]. Although these levels may decrease after biliary drainage or treatment of cholangitis, they may remain above the cutoff values used in the present study (CEA, 5.4 ng/mL; CA19-9, 89.4 U/mL) even when the elevation is not attributable solely to PHC. Therefore, the predictive value of tumor markers may be limited when they are considered in isolation. In contrast, combining these markers with age and NLR may provide a more comprehensive assessment of the risk of early recurrence. Indeed, patients with ≥3 points (HR-ER group) had a significantly higher 1-year recurrence rate than those with 0–2 points (NHR-ER group). Among the nine patients with exactly 3 points, four did not meet the age criterion, and five did not meet the CEA criterion, whereas all nine met both the CA19-9 and NLR criteria. Compared with existing preoperative prognostic models, the present risk classification specifically focuses on early recurrence within 1 year after curative-intent resection and uses four readily available preoperative factors in a simple, equally weighted scoring system. This approach may facilitate the identification of patients at increased risk of early recurrence without requiring complex calculations. However, because no direct comparison with existing models was performed, the incremental predictive value of this classification remains uncertain.

We also evaluated preoperatively assessable immune–nutritional and inflammatory markers in relation to early recurrence [26]. NLR, MLR, PLR, and SII were significantly higher in the HR-ER group, suggesting a greater degree of systemic inflammatory and immune imbalance in these patients. Cancer prognosis is closely associated with systemic inflammation and host immunity. Neutrophils and platelets may promote tumor progression through the release of inflammatory mediators and proangiogenic factors, whereas lymphocytes play an important role in antitumor immunity. Accordingly, elevated NLR and PLR may reflect an imbalance between tumor-promoting inflammation and impaired antitumor immune responses [27,28]. Similarly, an elevated MLR, reflecting increased monocyte-mediated inflammation and/or reduced lymphocyte-mediated antitumor immunity, has been associated with poor outcomes in patients with malignancies [29]. The SII, calculated using neutrophil, platelet, and lymphocyte counts, has been investigated as a marker of systemic inflammation and immune status [15].

Patients in the HR-ER group had significantly poorer DSS than those in the NHR-ER group. These findings suggest that systemic inflammatory status, particularly as reflected by NLR, may contribute to preoperative risk stratification for early recurrence. However, because the present risk classification was derived from a relatively small cohort, its prognostic utility and optimal cutoff values require validation in larger independent cohorts.

Several limitations should be considered when interpreting our findings. First, the retrospective design and relatively small sample size of this single-institution study may limit the generalizability of the results. We focused on objective, preoperatively assessable factors to identify predictors of early recurrence. However, intraoperative factors, postoperative pathological findings, and adjuvant chemotherapy may also influence the risk of early recurrence, and residual confounding by these factors cannot be excluded. In addition, four patients who died during the postoperative hospital stay before recurrence could be assessed were excluded from the analysis. Therefore, the findings primarily apply to patients who survived the postoperative hospitalization, and potential selection bias arising from this exclusion cannot be ruled out. Furthermore, the comparison of DSS between the ER and NER groups was based on postoperative recurrence status rather than baseline characteristics. Therefore, this analysis was descriptive and should not be interpreted as demonstrating a causal effect of early recurrence on survival. Second, the study included patients who underwent resection over a long period from 2007 to 2023, during which diagnostic and therapeutic strategies evolved. In particular, as described above, the indications for and regimens of preoperative treatment differed before and after 2011, resulting in heterogeneity in preoperative management. Furthermore, because laboratory parameters were assessed immediately before surgery, the tumor marker levels and inflammatory indices in patients who received neoadjuvant therapy may have been influenced by treatment-induced changes. Although surgery was generally performed after clinical resolution of cholangitis, residual inflammation may also have affected preoperative biomarker levels. These effects could not be fully accounted for and may have introduced residual confounding in the associations between preoperative laboratory parameters and early recurrence. Exploratory sensitivity analyses were performed among patients who did not receive neoadjuvant therapy, among those without preoperative cholangitis, and according to surgical period. The high-risk group showed higher estimated 1-year recurrence rates than the lower-risk group in all examined subgroups (Table S5). However, the number of high-risk patients was extremely small in each subgroup, limiting the ability of these analyses to establish the robustness of the proposed risk classification or exclude the potential influence of treatment exposure, cholangitis, and temporal changes in clinical practice. Moreover, changes in diagnostic imaging, surgical techniques, perioperative management, and neoadjuvant and adjuvant treatment strategies over the study period may limit the applicability of our findings to contemporary clinical practice. Although the exploratory analysis stratified by surgical period showed a similar direction of association between the proposed risk classification and early recurrence, the small number of high-risk patients precluded a reliable assessment of temporal consistency. Therefore, the generalizability of the identified preoperative predictors and the exploratory risk classification to patients treated under current multidisciplinary management strategies remains uncertain and should be evaluated in contemporary, independent multicenter cohorts. Third, the risk classification used to define the HR-ER and NHR-ER groups was developed and evaluated within the same relatively small cohort and has not been externally validated. Although bootstrap-based internal validation was performed, the optimism-corrected C-index was 0.668 when cutoff values were re-estimated within each bootstrap sample, indicating potential optimism in the apparent discriminatory performance. The limited number of early recurrence events and the data-driven selection of predictors and cutoff values raise concerns regarding potential overfitting. Furthermore, multicollinearity among the candidate inflammatory indices may have contributed to instability in predictor selection. The equal weighting of the four factors was adopted for simplicity and interpretability but was not formally optimized or validated. In addition, the high-risk group comprised only ten patients, resulting in substantial uncertainty in the estimated 1-year recurrence rate. The wide 95% confidence interval (42.2–92.9%) around the estimated 1-year recurrence rate of 70.0% highlights the limited precision of this estimate and the potential instability of the proposed risk classification. Moreover, the internal validation analyses did not reproduce the entire model development process, including predictor selection, cutoff determination, and score grouping, within a single bootstrap procedure. Therefore, the proposed risk classification should be considered exploratory and requires validation in larger independent cohorts. Accordingly, this risk classification should not be used to guide treatment decisions without further external validation. Furthermore, death without recurrence was not explicitly accounted for as a competing event in the conventional Cox proportional hazards and Kaplan–Meier analyses. Therefore, the estimated recurrence rates should be interpreted with caution, as they do not represent competing-risk-adjusted cumulative incidence estimates. Finally, PHC is a relatively rare disease, and the number of patients eligible for curative-intent resection is inherently limited [11,30,31]. Further multicenter studies with larger cohorts are needed to validate these findings and to better define preoperative risk stratification for early recurrence after curative-intent resection of PHC. Such studies should include independent cohorts from multiple institutions, with sufficient numbers of patients classified as HR-ER to allow more precise estimation of recurrence risk. The proposed cutoff values and risk-group definitions should be applied without modification, and discrimination, calibration, and recurrence rates with confidence intervals should be evaluated to assess the reproducibility and generalizability of the classification.

## 5. Conclusions

Early recurrence after curative-intent resection of PHC was associated with poor prognosis, highlighting the clinical importance of identifying patients at high risk of early recurrence before surgery. A risk classification based on four preoperatively assessable factors—age, CEA, CA19-9, and NLR—identified an HR-ER group with a 1-year recurrence rate of 70.0%. The high incidence of early recurrence in the HR-ER group highlights the need for strategies to reduce early recurrence. Whether intensified preoperative treatment and interventions targeting immune–nutritional status can reduce early recurrence and improve long-term outcomes in these high-risk patients should be investigated in future prospective studies. However, the proposed risk classification remains exploratory and should not be used to guide treatment decisions without external validation.

## Figures and Tables

**Table 1:** Comparison of Patient Characteristics Between ER and NER Groups.

Factors	ER (n = 24)	NER (n = 84)	*p*-Value
Age, years	66 (39–83)	71 (44–87)	**0.015**
Sex, male/female	15/9	59/25	0.472
Neoadjuvant therapy	14 (58.3%)	40 (47.6%)	0.355
PTPE	5 (20.8%)	14 (16.7%)	0.761
Preoperative cholangitis	11 (45.8%)	41 (48.8%)	0.797
Modified Glasgow Prognostic Score, 0/1/2	16/5/3	49/24/11	0.522
Preoperative ICGK	0.137 (0.083–0.233)	0.127 (0.076–0.309)	0.239
LHL15	0.940 (0.894–0.970)	0.950 (0.871–0.984)	0.203
Bismuth classification type IV	9 (37.5%)	25 (29.8%)	0.472
White blood cell, /μL	5390 (3080–9310)	5120 (2580–8400)	0.485
Neutrophil, /μL	3350 (1550–5150)	2950 (1130–5970)	0.227
Lymphocyte, /μL	1380 (530–3140)	1520 (540–2530)	0.100
Monocyte, /μL	460 (190–921)	450 (190–960)	0.837
Platelet, 10^4^/μL	22.2 (11.5–72.4)	21.5 (10.0–40.4)	0.431
Total protein, g/dL	6.8 (5.7–7.9)	6.9 (5.4–8.3)	0.665
Albumin, g/dL	3.8 (3.0–4.4)	3.7 (3.0–4.8)	0.467
CRP, mg/dL	0.26 (0.03–2.73)	0.23 (0.01–3.97)	0.646
Total bilirubin, mg/dL	0.8 (0.0–2.1)	0.8 (0.3–3.4)	0.835
Carcinoembryonic antigen, ng/mL	3.0 (0.9–32.3)	2.7 (0.5–20.0)	0.261
Carbohydrate antigen 19-9, U/mL	94 (0–4703)	56 (1–1890)	0.276
Neutrophil-to-lymphocyte ratio	2.25 (0.66–8.15)	2.02 (0.63–6.53)	0.088
Monocyte-to-lymphocyte ratio	0.29 (0.17–1.15)	0.29 (0.11–0.91)	0.646
Platelet-to-lymphocyte ratio	172 (82–680)	149 (51–459)	0.078
Prognostic nutritional index	44.8 (32.7–55.7)	45.3 (35.4–55.6)	0.859
Controlling Nutritional Status score	1.5 (0–6)	2 (0–7)	0.936
CRP-to-albumin ratio, mg/dL per g/dL	0.07 (0.01–0.86)	0.06 (0.00–1.24)	0.662
CALLY index	1.90 (0.06–36.26)	2.47 (0.07–36.55)	0.494
Systemic immune–inflammation index	576 (197–3046)	429 (84–2640)	0.079
Systemic inflammation response index	1.05 (0.26–5.32)	0.86 (0.21–3.54)	0.260
Clinical T classification before surgery, 1/2/3/4	0/2/7/15	3/30/24/27	**0.002**
Clinical N classification before surgery, 0/1 or 2	16/8	64/20	0.348

CALLY, C-reactive protein–albumin–lymphocyte index; CRP, C-reactive protein; ER, early recurrence; ICGK, k-value of the indocyanine green test; LHL15, liver-to-heart ratio at 15 min; NER, non-early recurrence; PTPE, percutaneous transhepatic portal vein embolization. Data are expressed as numbers (percentages) or medians (ranges). Bold values indicate statistical significance (*p* < 0.05).

**Table 2:** Univariable and Multivariable Analysis of Risk Factors for Early Recurrence.

Factors	Univariable Analysis		Multivariable Analysis	
	Hazard Ratio (95% CI)	*p*-Value	Hazard Ratio (95% CI)	*p*-Value
Age	0.951 (0.916–0.989)	**0.011**	0.942 (0.901–0.985)	**0.008**
Male sex	1.438 (0.629–3.287)	0.389		
Neoadjuvant therapy	1.424 (0.633–3.207)	0.393		
PTPE	1.488 (0.555–3.986)	0.430		
Preoperative cholangitis	0.900 (0.403–2.008)	0.796		
Preoperative ICGK	1.064 (0.012–92.701)	0.978		
LHL15	0.000 (0.000–4147.123)	0.314		
Bismuth classification type IV	1.349 (0.590–3.083)	0.478		
White blood cell	1.000 (1.000–1.000)	0.465		
Neutrophil	1.000 (1.000–1.000)	0.251		
Lymphocyte	0.999 (0.998–1.000)	0.158		
Monocyte	1.001 (0.998–1.003)	0.543		
Platelet	1.038 (0.999–1.077)	0.054		
Total protein	0.939 (0.498–1.770)	0.845		
Albumin	1.298 (0.481–3.501)	0.607		
CRP	1.097 (0.728–1.651)	0.659		
Total bilirubin	0.941 (0.401–2.208)	0.888		
Carcinoembryonic antigen	1.104 (1.057–1.153)	**<0.001**	1.115 (1.056–1.177)	**<0.001**
Carbohydrate antigen 19-9	1.001 (1.000–1.001)	**<0.001**	1.001 (1.000–1.001)	**0.024**
Modified Glasgow prognostic score				
1 vs. 0	0.679 (0.249–1.853)	0.449		
2 vs. 0	0.893 (0.260–3.065)	0.857		
Neutrophil-to-lymphocyte ratio	1.406 (1.068–1.850)	**0.015**	1.404 (1.035–1.905)	**0.029**
Monocyte-to-lymphocyte ratio	5.428 (0.744–39.582)	0.095		
Platelet-to-lymphocyte ratio	1.006 (1.002–1.009)	**0.003**	-	
Prognostic nutritional index	0.994 (0.911–1.085)	0.894		
Controlling Nutritional Status score	1.047 (0.813–1.350)	0.720		
CRP-to-albumin ratio	1.264 (0.306–5.213)	0.746		
CALLY index	0.984 (0.925–1.048)	0.621		
Systemic immune–inflammation index	1.001 (1.000–1.002)	**0.003**	-	
Systemic inflammation response index	1.447 (1.008–2.076)	**0.045**	-	
Clinical T classification before surgery				
(y)cT3-4 vs. 1-2	5.791 (1.361–24.628)	**0.017**	-	
Clinical N classification before surgery				
(y)cN1-2 vs. 0	1.544 (0.661–3.608)	0.316		

CALLY, C-reactive protein–albumin–lymphocyte index; CI, confidence interval; CRP, C-reactive protein; ICGK, k-value of the indocyanine green test; LHL15, liver-to-heart ratio at 15 min; PTPE, percutaneous transhepatic portal vein embolization. Bold values indicate statistical significance (*p* < 0.05).

**Table 3:** Comparison of Clinicopathological Features Between the High-Risk Early Recurrence Group (HR-ER) and the Non-High-Risk Early Recurrence Group (NHR-ER).

Factors	HR-ER (n = 10)	NHR-ER (n = 95)	*p*-Value
Age, years	59.5 (39–80)	70 (44–87)	**0.007**
Sex, male/female	6/4	68/27	0.477
Neoadjuvant therapy performed	6 (60.0%)	45 (47.4%)	0.519
PTPE	3 (30.0%)	16 (16.8%)	0.383
Preoperative cholangitis	8 (80.0%)	44 (46.3%)	0.052
Modified Glasgow Prognostic Score, 0/1/2	4/4/2	58/25/12	0.313
Preoperative ICGK	0.121 (0.086–0.170)	0.134 (0.076–0.309)	0.601
LHL15	0.948 (0.920–0.970)	0.948 (0.871–0.984)	0.919
Bismuth classification type IV	5 (50.0%)	28 (29.5%)	0.281
White blood cell, /μL	6295 (3610–8090)	5100 (2580–9310)	**0.038**
Neutrophil, /μL	4095 (2290–5040)	2900 (1130–5970)	**0.013**
Lymphocyte, /μL	1355 (530–1770)	1500 (540–3140)	0.172
Monocyte, /μL	500 (190–921)	445 (190–960)	0.488
Platelet, 10^4^/μL	25.0 (11.5–72.4)	21.7 (10.0–40.4)	0.268
Total protein, g/dL	6.6 (6.0–8.2)	6.8 (5.4–8.1)	0.537
Albumin, g/dL	3.7 (3.0–4.3)	3.8 (3.0–4.8)	0.634
CRP, mg/dL	0.45 (0.08–2.73)	0.23 (0.01–3.97)	0.234
Total bilirubin, mg/dL	0.9 (0.3–1.8)	0.8 (0.0–3.4)	0.673
Carcinoembryonic antigen, ng/mL	4.7 (1.1–30.8)	2.6 (0.5–32.3)	0.095
Carbohydrate antigen 19-9, U/mL	273 (89–4703)	47 (0–1890)	**<0.001**
Neutrophil-to-lymphocyte ratio	3.41 (2.06–5.01)	2.04 (0.63–8.15)	**0.002**
Monocyte-to-lymphocyte ratio	0.40 (0.21–0.54)	0.28 (0.11–1.15)	**0.037**
Platelet-to-lymphocyte ratio	219 (95–425)	149 (51–680)	**0.012**
Prognostic nutritional index	43.6 (32.7–45.6)	45.6 (35.4–55.7)	0.404
Controlling Nutritional Status score	2 (1–6)	2 (0–7)	0.796
CRP-to-albumin ratio, mg/dL per g/dL	0.12 (0.02–0.86)	0.06 (0.00–1.24)	0.219
CALLY index	1.34 (0.06–5.85)	2.53 (0.07–36.55)	0.147
Systemic immune–inflammation index	752 (480–2053)	421 (84–3046)	**0.001**
Systemic inflammation response index	1.40 (0.82–3.54)	0.86 (0.21–5.32)	**0.005**
Portal vein resection and reconstruction	7 (70.0%)	33 (34.7%)	**0.041**
Hepatic artery resection and reconstruction	4 (40.0%)	18 (18.9%)	0.212
Operative time, min	620 (462–972)	606 (395–1062)	0.435
Blood loss, mL	3189 (369–6012)	1443 (101–6916)	**0.005**
Clavien-Dindo classification ≥ IIIa	4 (40.0%)	55 (57.9%)	0.328
**Pathological T classification**			
no residual tumor/T1/T2/T3/T4	0/0/4/1/5	2/11/51/17/14	0.134
Pathological N classification, 1 or 2	9 (90.0%)	34 (35.8%)	**0.001**
Poorly differentiated adenocarcinoma	1 (10.0%)	9 (9.5%)	1.000
Adjuvant chemotherapy performed	9 (90.0%)	73 (76.8%)	0.687
Postoperative hospital stay, day	42 (17–117)	40 (13–161)	0.639

CALLY, C-reactive protein–albumin–lymphocyte index; CRP, C-reactive protein; ICGK, k-value of the indocyanine green test; LHL15, liver-to-heart ratio at 15 min; PTPE, percutaneous transhepatic portal vein embolization. Data are expressed as numbers (percentages) or medians (ranges). Bold values indicate statistical significance (*p* < 0.05).

## Data Availability

The datasets generated and analyzed during the current study are available from the corresponding author upon reasonable request.

## Supplementary Materials

The following supporting information can be downloaded at: https://www.mdpi.com/article/10.3390/cancers18213568/s1, Table S1: Surgical characteristics, postoperative treatment, and recurrence patterns according to early recurrence status; Table S2: Variance inflation factors for the eight candidate variables and the four variables retained in the final Cox model; Table S3: Complete-case multivariable Cox proportional hazards analysis of early recurrence; Table S4: Clinicopathological characteristics according to age (<61 vs. ≥61 years); Table S5: Association between the preoperative risk classification and early recurrence in exploratory subgroup analyses.

## Abbreviations

The following abbreviations are used in this manuscript:

PHC	Perihilar cholangiocarcinoma
ER	Early recurrence
NER	Non-early recurrence
HR-ER	High-risk early recurrence
NHR-ER	Non-high-risk early recurrence
CEA	Carcinoembryonic antigen
CA19-9	Carbohydrate antigen 19-9
NLR	Neutrophil-to-lymphocyte ratio
MLR	Monocyte-to-lymphocyte ratio
PLR	Platelet-to-lymphocyte ratio
CAR	C-reactive protein-to-albumin ratio
PNI	Prognostic nutritional index
CONUT	Controlling nutritional status
CALLY	C-reactive protein–albumin–lymphocyte
SII	Systemic immune–inflammation index
SIRI	Systemic inflammation response index
RFP	Recurrence-free probability
DSS	Disease-specific survival
ROC	Receiver operating characteristic
