# Supplementary material for: Preoperative Risk Classification for Early Recurrence After Curative-Intent Resection of Perihilar Cholangiocarcinoma: A Single-Center Retrospective Study
Source: Cancers (Basel). 2026 Nov 6;18(21):3568. doi: 10.3390/cancers18213568 (PMC13762085; doi:10.3390/cancers18213568)
Supplement: Supplementary file 1 [file cancers-18-03568-s001.zip › Supplementary Table S1.pdf]

Table S1. Surgical characteristics, postoperative treatment, and recurrence patterns according to early recurrence status

Factors	ER (n = 24)	NER (n = 84)
**Surgical procedure**		
Hepatopancreatoduodenectomy (HPD)	1 (4.2%)	3 (3.6%)
Hepatic trisectionectomy	5 (20.8%)	4 (4.8%)
Other procedures	18 (75.0%)	77 (91.7%)
Positive resection margin	9 (37.5%)	20 (23.8%)
Clavien–Dindo classification ≥ IIIa	12 (50.0%)	48 (57.1%)
Adjuvant chemotherapy	20 (83.3%)	64 (76.2%)
**Recurrence patterns**		
Patients with recurrence	24 (100%)	33 (39.3%)
Local recurrence	7 (29.2%)	15 (45.5%)
Lymph nodes	8 (33.3%)	5 (15.2%)
Lung	3 (12.5%)	4 (12.1%)
Liver	6 (25.0%)	7 (21.2%)
Peritoneal dissemination	5 (20.8%)	5 (15.2%)
Bone	1 (4.2%)	0 (0.0%)

Data are presented as n (%). ER, early recurrence; NER, non-early recurrence; HPD, hepatopancreatoduodenectomy. Percentages for surgical characteristics, postoperative treatment, and patients with recurrence were calculated using all patients in each group (ER, n = 24; NER, n = 84). Percentages for recurrence sites were calculated among patients with documented recurrence (ER, n = 24; NER, n = 33). Recurrence sites were not mutually exclusive; patients with recurrence at multiple sites were counted in each applicable category. The NER group included patients with recurrence more than 12 months after surgery and patients without recurrence.
