# Supplementary material for: Preoperative Risk Classification for Early Recurrence After Curative-Intent Resection of Perihilar Cholangiocarcinoma: A Single-Center Retrospective Study
Source: Cancers (Basel). 2026 Nov 6;18(21):3568. doi: 10.3390/cancers18213568 (PMC13762085; doi:10.3390/cancers18213568)
Supplement: Supplementary file 1 [file cancers-18-03568-s001.zip › Supplementary Table S2.pdf]

Table S2. Variance inflation factors for the eight candidate variables and the four variables retained in the final Cox model.

Variable	Eight candidate variables	Final four-variable model
Age	1.142	1.038
CEA	1.224	1.134
CA19-9	1.189	1.115
NLR	3.543	1.019
PLR	4.986	—
SII	8.798	—
SIRI	2.507	—
Clinical T classification	1.073	—

VIF, variance inflation factor; CEA, carcinoembryonic antigen; CA19-9, carbohydrate antigen 19-9; NLR, neutrophil-to-lymphocyte ratio; PLR, platelet-to-lymphocyte ratio; SII, systemic immune-inflammation index; SIRI, systemic inflammation response index.

VIFs were calculated using the same complete-case cohort (n = 98) for both models. A dash indicates that the variable was not retained in the final four-variable model.
