# Supplementary material for: Preoperative Risk Classification for Early Recurrence After Curative-Intent Resection of Perihilar Cholangiocarcinoma: A Single-Center Retrospective Study
Source: Cancers (Basel). 2026 Nov 6;18(21):3568. doi: 10.3390/cancers18213568 (PMC13762085; doi:10.3390/cancers18213568)
Supplement: Supplementary file 1 [file cancers-18-03568-s001.zip › Supplementary Table S3.pdf]

Table S3. Complete-case multivariable Cox proportional hazards analysis of early recurrence

Variable	Hazard ratio	95% confidence interval	p-value
Age	0.936	0.895–0.978	0.003
CEA	1.121	1.062–1.184	<0.001
CA19-9	1.0005	1.0001–1.0010	0.022
NLR	1.362	1.003–1.851	0.048

Complete-case analysis included 105 patients with available data for age, CEA, CA19-9, and NLR, including 23 patients with early recurrence. All four predictors were entered simultaneously into the multivariable Cox proportional hazards model without stepwise variable selection.

Abbreviations: CEA, carcinoembryonic antigen; CA19-9, carbohydrate antigen 19-9; NLR, neutrophil-to-lymphocyte ratio.
