# Supplementary material for: Preoperative Risk Classification for Early Recurrence After Curative-Intent Resection of Perihilar Cholangiocarcinoma: A Single-Center Retrospective Study
Source: Cancers (Basel). 2026 Nov 6;18(21):3568. doi: 10.3390/cancers18213568 (PMC13762085; doi:10.3390/cancers18213568)
Supplement: Supplementary file 1 [file cancers-18-03568-s001.zip › Supplementary Table S4.pdf]

Table S4. Clinicopathological characteristics according to age (<61 vs. ≥61 years).

Factors	Age <61 (n = 17)	Age ≥61 (n = 91)	p-value
Neoadjuvant therapy	13 (76.5%)	41 (45.1%)	**0.032**
Bismuth classification type IV	7 (41.2%)	27 (29.7%)	0.398
Neutrophil-to-lymphocyte ratio	2.66 (1.03-5.91)	2.02 (0.63-8.15)	0.039
Carcinoembryonic antigen, ng/mL	2.1 (0.5-5.4)	3.0 (0.8-32.3)	0.057
Carbohydrate antigen 19-9, U/mL	54 (0-358)	60 (1-4703)	0.309
HPD or hepatic trisectionectomy	3 (17.6%)	10 (11.0%)	0.427
Portal vein resection and reconstruction	11 (64.7%)	31 (34.1%)	**0.028**
Hepatic artery resection and reconstruction	5 (29.4%)	17 (18.7%)	0.332
Operative time, min	633 (462-972)	605 (383-1062)	0.074
Blood loss, mL	1777 (369-6916)	1443 (101-6059)	0.114
Clavien–Dindo classification ≥ IIIa	10 (58.8%)	50 (54.9%)	0.797
**Pathological T classification**			
no residual tumor/ T1/ T2/ T3/ T4	1/ 3/ 3/ 3/ 7	1/ 8/ 53/ 16/ 13	**0.004**
Pathological N classification, 1 or 2	8 (47.1%)	37 (40.7%)	0.789
Adjuvant chemotherapy performed	13 (76.5%)	71 (78.0%)	1.000
Postoperative hospital stay, day	45 (13-128)	39 (13-161)	0.613

The analysis included all 108 patients.

Data are presented as n (%) for categorical variables and median (range) for continuous variables.

All percentages are calculated using the respective age-group denominators (n = 17 and n = 91).

HPD, hepatopancreatoduodenectomy.
