# Supplementary material for: Preoperative Risk Classification for Early Recurrence After Curative-Intent Resection of Perihilar Cholangiocarcinoma: A Single-Center Retrospective Study
Source: Cancers (Basel). 2026 Nov 6;18(21):3568. doi: 10.3390/cancers18213568 (PMC13762085; doi:10.3390/cancers18213568)
Supplement: Supplementary file 1 [file cancers-18-03568-s001.zip › Supplementary Table S5.pdf]

Table S5. Association between the preoperative risk classification and early recurrence in exploratory subgroup analyses

Subgroup	Risk group	Patients, n	ER, n	1-year recurrence rate	p-value
No neoadjuvant therapy	NHR-ER	50	7	14.5%	<0.001
(n = 54)	HR-ER	4	3	75.0%	
No preoperative cholangitis	NHR-ER	51	10	20.4%	<0.001
(n = 53)	HR-ER	2	2	100.0%	
2007–2015	NHR-ER	37	8	21.8%	0.20
(n = 43)	HR-ER	6	3	50.0%	
2016–2023	NHR-ER	58	8	14.4%	<0.001
(n = 62)	HR-ER	4	4	100.0%	

NHR-ER, non-high-risk early recurrence group (0–2 points); HR-ER, high-risk early recurrence group (3–4 points); ER, early recurrence. Estimated 1-year recurrence rates were calculated as one minus the Kaplan–Meier estimate of recurrence-free probability. Between-group differences were assessed using the log-rank test. Patients who died without documented recurrence were censored at death. Analyses were restricted to patients with complete risk-score data (n = 105). The no-neoadjuvant-therapy and no-preoperative-cholangitis analyses were restricted to the respective subgroups. Because of the small high-risk subgroup sizes, these analyses are exploratory.
